# Cold shock induction of recombinant Arctic environmental genes

**DOI:** 10.1186/s12896-015-0185-1

**Published:** 2015-08-19

**Authors:** Gro Elin Kjæreng Bjerga, Adele Kim Williamson

**Affiliations:** Norstruct, Department of Chemistry, Faculty of Science and Technology, University of Tromsø, N-9037 Tromsø, Norway; Centre for Applied Biotechnology, Uni Research AS, Thormøhlensgt. 55, N-5008 Bergen, Norway

**Keywords:** *cspA* promoter, Heterologous expression, Fusion partner, Restriction-free cloning, Cold-adapted, Metagenomic DNA

## Abstract

**Background:**

Heterologous expression of psychrophilic enzymes in *E. coli* is particularly challenging due to their intrinsic instability. The low stability is regarded as a consequence of adaptation that allow them to function at low temperatures. Recombinant production presents a significant barrier to their exploitation for commercial applications in industry.

**Methods:**

As part of an enzyme discovery project we have investigated the utility of a cold-shock inducible promoter for low-temperature expression of five diverse genes derived from the metagenomes of marine Arctic sediments. After evaluation of their production, we further optimized for soluble production by building a vector suite from which the environmental genes could be expressed as fusions with solubility tags.

**Results:**

We found that the low-temperature optimized system produced high expression levels for all putatively cold-active proteins, as well as reducing host toxicity for several candidates. As a proof of concept, activity assays with one of the candidates, a putative chitinase, showed that functional protein was obtained using the low-temperature optimized vector suite.

**Conclusions:**

We conclude that a cold-shock inducible system is advantageous for the heterologous expression of psychrophilic proteins, and may also be useful for expression of toxic mesophilic and thermophilic proteins where properties of the proteins are deleterious to the host cell growth.

**Electronic supplementary material:**

The online version of this article (doi:10.1186/s12896-015-0185-1) contains supplementary material, which is available to authorized users.

## Background

Enzymes used in industry often originate from extremophilic organisms to fit the operating conditions for biocatalysis required by the process [1, 2]. These enzymes have evolved to function in ecological niches that may include very low or very high temperature, extreme pH, high salt concentration or high pressure. Recombinant production of some of these enzymes presents a significant challenge for their use in biotechnology and industry due to the demands of these applications for large amounts of highly pure protein [2, 3]. This is particularly relevant in the case of cold-adapted (psychrophilic) enzymes [4, 5] as the intrinsic instability of these proteins, which is a consequence of adaptations that allow them to function at low temperatures, makes them more challenging to produce than their mesophilic and thermophilic homologues (reviewed in [6, 7]). Little attention has been given to heterologous expression of psychrophilic enzymes, despite their large range of technological applications and commercial value (reviewed in [8]). In this study, we present a vector suite designed to optimize *E. coli*-based expression of putatively cold-adapted proteins which are encoded by genes sourced from the marine Arctic environment. We have opted to control the expression of our recombinant proteins by a cold-shock inducible promoter, the *E. coli* cold-shock protein A (*cspA)* promoter [9, 10]. This promoter drives high levels of protein expression, comparable to the commonly used *T*7-system, but is induced by a downshift in temperature rather than addition of an exogenous chemical inducer [9, 10]. In addition, we have utilized several well-documented solubilization tags, such as the maltose-binding protein (MBP) [11–13], the nascent chain chaperone trigger factor (TF) [14, 15], *E. coli* thioredoxin (TRX) [16] and small ubiquitin-like modifier (SUMO) [17] as fusion partners to our candidate proteins, combined with the hexahistidine-tag (His) for purification [18–21]. Using a restriction-free (RF) cloning method [22, 23], we have built up a suite of vectors encoding these fusion partners, based on the pCold-II vector that encodes the *cspA* promoter [24]. This vector suite facilitates the parallel testing of several important factors for recombinant expression, solubility and functionality of putatively cold-active enzymes encoded by environmental genes of Arctic origin.

## Methods

### Origins and cloning of candidate genes

The genes used in this study are part of a large bioprospecting project (the Norwegian MARZymes initiative), which aims to discover and develop commercially useful, cold-active enzymes sourced from the Arctic environment. Fosmid libraries were generated from metagenomic DNA purified from two sediment samples that were collected from either the sea floor of the Barents Sea (candidates MZ0003, MZ0009, MZ0012, MZ0013), or the intertidal zone at Kapp Wijk, Svalbard (MZ0047). The details of library preparation are outside of the scope of this publication, and in the case of the Kapp Wijk sample are described elsewhere [25]. The Fosmid inserts were sequenced using 454 technology [26], and the relevant gene sequences have been deposited to the European Nucleotide Archive [ENA: LM651246, LM651247, LM651248, LM651249 and LM651250]. Open reading frames were found in the Fosmid sequences using the MetaGeneMark prediction tool [27]. Gene candidates and putative activities were identified based on homology searches using t-BLAST [28]. Sequences were analyzed for the presence of signal peptides, which would target trafficking across the cytoplasmic membrane in the native host, by the SignalP 4.1 server [29] using the default cutoff values. Both Gram-positive and Gram-negative organism settings were run as the species these genes originate from are unknown. Domain information was assigned using the Pfam database with a threshold E-value of 1.0 [30]. Structural homologues and predicted tertiary structures were analyzed using the homology modeling software SWISS-MODEL [31] in the ‘automodel’ mode*.* The molecular weight and pI were calculated using the ProtParam tool at the ExPASy server [32].

The candidate genes described in this paper represent a sub-set of ‘difficult’ targets from the bioprospecting project, which had previously failed in recombinant expression trials using *T*7-based promoter systems. Previously, the genes were cloned as constructs with a variety of truncations, signal sequences or fusion tags and then expressed in various cell strains at different temperatures in attempts to obtain sufficient amounts of soluble product for characterization (Additional file 1: Table S1–S3). Truncations were designed to express individual fragments or domains annotated from Pfam or trimmed to remove predicted signal peptides. The reasons for failed expression included insoluble production as inclusion bodies or toxicity resulting in death of the expression cultures (Additional file 1: Table S1–S3). Sub-cloning of genes into the pCold-II-based vectors was done by RF-cloning [22, 23], as described below. Several of the candidates were truncated based on the above-mentioned bioinformatic analyses of the sequences, our previous examples of successful *T*7-based expression (Additional file 1: Table S1–S3), and on the observation that N-terminal and C-terminal truncations can reduce flexibility and increase stability [33]. The regions cloned are listed in Table 1.

**Table 1:** Summary of *in silico* analysis and cloning information of candidate sequences

Candidate	Putative activity	tBlast homologue (% identity)^a^	PDB homologue (residue range in aa, % identity)^b^	Rare codons (%)^c^	SignalP prediction result for Gram negative/Gram positive (cleavage position)	Molecular mass native/recombinant (Kda)	European nucleotide archive accession number
MZ0003	Carbohydrate esterase Family 15	Acetyl xylan esterase *Planctomyces maris* (57)	3picB (39–430, 20.8)	6	N/Y(25–26)	48/46	LM651246
MZ0009	Glycosyl hydrolases family 18	Glycoside hydrolase *Herpetosiphon aurantiacus* DSM 785 (26)	3g6mA (27–358, 23.0)	12	N/Y(28–29)	48/36	LM651247
MZ0012	Glycosyl hydrolases family 39	β-xylosidase *Mycobacterium abscessus* (42)	4ekjA (65–358, 26.3)	13	N/N	42/42	LM651248
MZ0013	Carbohydrate esterase Family 4	Polysaccharide deacetylase *Halorhodospira halophila* SL1 (64)	3vus2A (31–259, 26.4)	6	Y/Y(30–31)	39/21	LM651249
MZ0047	ATP dependent DNA ligase	ATP dependent DNA ligase *Marinobacter adhaerens* HP15 (70)	4d05A (39–287, 46.6)	8	Y(33–34)/Y(31–32)	32/29	LM651250

^a^Based on Pfam and NCBI conserved domain annotations and BLAST homology

^b^Determined from SwissModel structure homology

^c^Determined from the Codon Frequency Distribution analysis tool (GenScript) and includes codons with values lower than 30 that are likely to hamper the expression efficiency

### Construction of a pCold-II vector suite

To enable tag-removal at a later stage of purification, a Tobacco Etch Virus (TEV) protease site was introduced to the pCold-II vector (Takara) by annealed oligonucleotide cloning. 50 μM of each of the oligonucleotides TEV5’ and TEV3’ (Additional file 1: Table S4) were hybridized at 95 °C for 5 min, and cooled to 4 °C. 0.75 μM of the annealed oligos were phosphorylated by 10 U T4 polynucleotide kinase (NEB) in T4 kinase buffer supplemented with 1 mM ATP (Sigma). After stopping the reaction with 20 mM EDTA, 0.5 pmols annealed oligos were ligated in a 1:10 ratio of oligos to *Bam*HI (NEB) and CIAP (Finnzymes) treated pCold-II vector, using T4 DNA ligase (NEB). Vectors encoding different fusion partners were generated by introducing genes for the solubility tags between the existing His-tag and TEV site of the pCold-II-TEV vector using RF-cloning [22, 23, 34]. Genes encoding the TRX-, MBP-, TF- and SUMO-tags were amplified from templates using the primers listed in Additional file 1: Table S4 that were designed using an online tool described in [35]. The *E. coli trx* gene encoding residues 2–109 of TRX [UniProt: P0AA25] was amplified from pET32a(+) (Novagen). The *E. coli mal*E gene encoding residues 27–391 of MBP [UniProt: P0AEX9] was amplified from pHMGWA [36]. Genomic DNA isolated from a DH5α strain was template for the *E. coli tig* gene encoding residues 2–432 of TF [UniProt: B7UJQ9] and a codon-optimized yeast *smt3* gene (GenScript) served as template for SUMO [UniProt: Q12306] encoding resides 2–98. The megaprimers containing fusion partner genes were amplified using Phusion polymerase (Finnzymes), purified with the QIAquick PCR purification kit (Qiagen) and inserted in the vector by linear plasmid amplification. To remove parental DNA, the PCR products were digested with 20 U *Dpn*I (NEB) and transformed to *E. coli* XL1blue cells by conventional heat shock. Sanger sequencing was used to confirm correct cloning of all vectors. Information about the vectors used in this study is summarized in Table 2.

**Table 2:** Expression vectors used in this study

Vector	Fusion partners	Fusion partner size (kDa)^a^	Promoter	Linker (aa)^b^	Resistance gene	Manufacturer
pCold-II	His	n.d.	*cspA*	-	*bla*	Takara
pCold-II-TEV	His	3	*cspA*	16	*bla*	This study
pCold-II-TRX-TEV	His, TRX	15	*cspA*	11	*bla*	This study
pCold-II-MBP-TEV	His, MBP	43	*cspA*	11	*bla*	This study
pCold-II-SUMO	His, SUMO	13	*cspA*	0	*bla*	This study
pCold-II-TF-TEV	His, TF	51	*cspA*	12	*bla*	This study

^a^ *n.d.* not determined (not relevant for this work)

^b^Given as the region from the His-tag to the start of candidate sequence insertion site, including the TEV site (7 aa)

### Insertion of candidate genes into the pCold-II vector suite

To introduce candidate genes into the panel of pCold-II vectors, megaprimers of each candidate were generated by PCR and then inserted into vectors by RF-cloning as described above. Clone selection was carried out by PCR screening of single colonies using a *Taq* DNA polymerase master mix (Ampliqon). Plasmids were isolated using the Wizard plasmid purification kit (Promega), and Sanger sequencing confirmed successful insertions of sequences to vectors.

### Recombinant protein expression

Recombinant proteins were expressed in 24 deep well plates (DW24) according to the protocol described in [37]. Plasmids were transformed into the *E. coli* host strains, Rosetta2(DE3)pLysS, BL21CodonPlus(DE3)RIL or ArcticExpress(DE3)RIL by heat shock. Cells were grown in 4 ml 2YT medium supplemented with 2 % (w/v) D-glucose and relevant antibiotics (100 μg/ml ampicillin, 25 μg/ml kanamycin, 12.5 μg/ml tetracycline, 20 μg/ml gentamycin and/or 34 μg/ml chloramphenicol) at 37 °C in a Micro-Expression Plate Shaker (GlasCol) at 450 rpm for 2–4 h until cells reached optical density at 600 nm (OD_600_) of 0.5–0.9. Recombinant expression was induced by a temperature downshift to 15 °C and addition of 0.4 mM IPTG. After 16–20 h cells were harvested by centrifugation and resuspended in 1 ml of 50 mM TrisHCl pH 8.0, 250 mM NaCl, 1x cOmplete protease inhibitor cocktail (Roche) and lysed by three 5 s pulses at 25 % amplitude on VibraCell sonicator (Sonics) with a 3 mm microtip. Samples collected before and after a centrifugation at 3461 x* g* for 30 min were analyzed on 4–20 % MiniProtean TGX precast SDS-PAGE gels (BioRad) to determine the total amounts of induced and soluble protein, respectively. Successful expression of recombinant protein was assessed as the presence of a band in the post-induction sample at the molecular weight predicted by its primary sequence, and was scored on a qualitative scale from 0 to 3: 0, no visible recombinant protein; 1, visible recombinant protein at similar levels as endogenous host proteins (<10 % of total); 2, visible recombinant protein at higher levels than endogenous host proteins (≈10–50 % of total); and 3, visible recombinant protein at very high levels (>50 % of total). Failure of the cells to grow after transformation was taken as a sign of toxicity, and these samples were assigned a score of −1. To determine the solubility of recombinant proteins, the density of the protein bands was quantified by integration of the band intensity to area from SDS-PAGE gels after Coomassie staining using the QuantityOne software (BioRad) and normalized to the culture OD_600_ at harvest to correct for different growth rates. Solubility was calculated as a percentage of the total expression yield after subtraction of background intensity.

### Chitinase assay

To measure chitinase activity, a fluorometric assay (Sigma) was performed according to the manufacturer’s instructions. Activity from 10 μl of crude extract was assayed with 0.72 μg substrate in 0.1 M Na_2_HPO_4_ at 20 °C in 100 μl final volume. Three different chemicals, all labeled with 4-methylumbelliferone (4MU), 4MU-*N*-acetyl-β-D-glucosaminide, 4MU-*N*,*N*’-diacetyl-β-D-chitobioside and 4MU-β-D-*N*,*N*’,*N*”-triacetylchitotriose, were used as substrate analogs of chitin. Reactions were stopped with 1 M alkaline sodium carbonate. The release of fluorometric 4MU was measured using an excitation wavelength of 360 nm and emission wavelength of 450 nm in a SpectroMax microplate reader (Molecular Devices). The background fluorescence intensities (relative values) were subtracted form the experimental values, which were then normalized to the semi-quantitative measure of soluble protein (as explained above). A chitinase extract from *Trichoderma viride* (Sigma) was used as a positive control.

## Results and discussion

### Selection of candidates and rationale for their recombinant expression

**Fig. 1:**
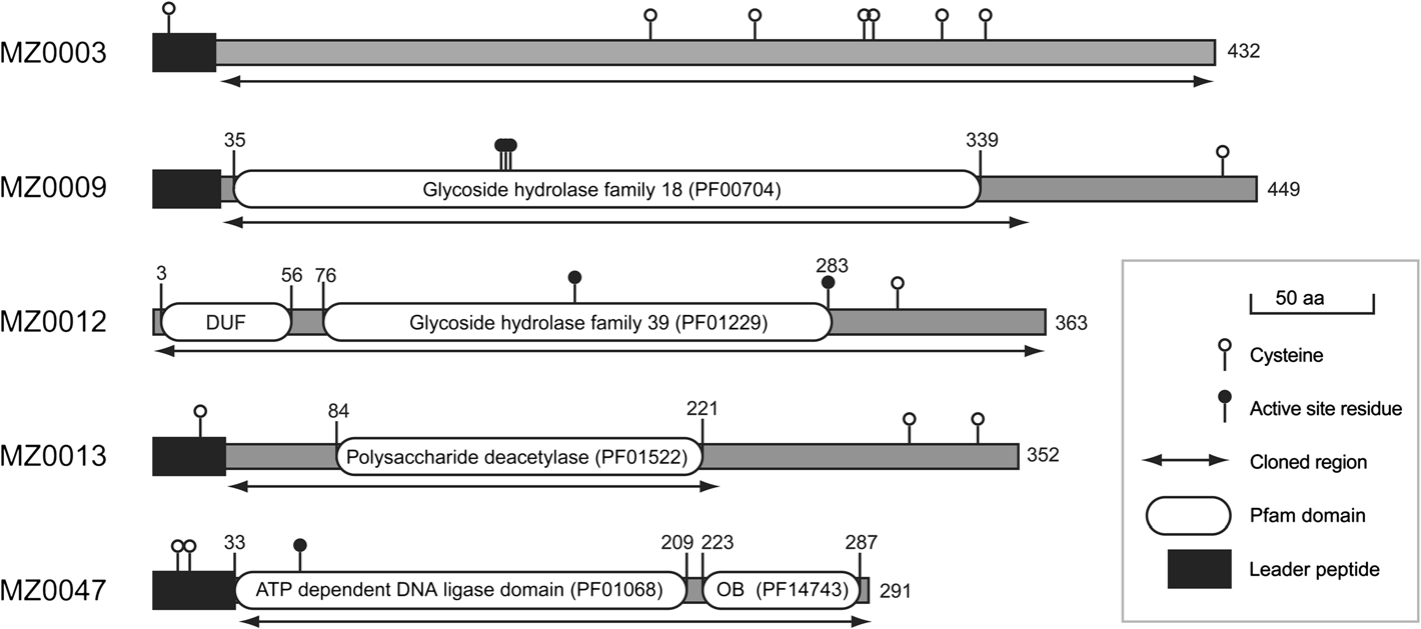
Environmental candidate sequences. Cartoon of the sequences of all candidates annotated with leader sequences (*black boxes*) and predicted Pfam domains (*white boxes*, borders are given by residue number), drawn to scale. No Pfam domain predictions were found for MZ0003. The presence of known catalytic residues and cysteines is highlighted with black and white circles, respectively. The length of native proteins are given next to each candidate, and cloned regions are indicated with arrowed lines

**Fig. 2:**
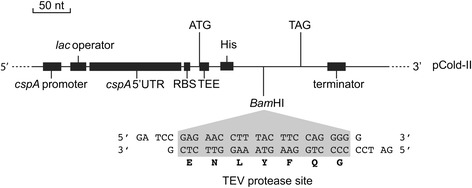
Cloning and expression region of pCold-II-TEV. The cold-shock protein A promoter (*cspA*), the *lac* operator, ribosome binding site (RBS) and the translation-enhancing element (TEE) regulate gene expression from pCold-based vectors. The *cspA*-derived 5′UTR region allows low-temperature induction as it is stable at temperatures around 15 °C, but highly unstable at 37 °C. A TEV protease recognition sequence was introduced at the *Bam*HI restriction site of the cloning region of pCold-II to allow removal of the vector-encoded His-tag from recombinant proteins. The cartoon is drawn to scale

The genes tested in this study are part of a bioprospecting project that aims to discover new enzymes with potential commercial applications, and includes two putative carbohydrate esterases, two putative glycosyl hydrolases and a putative ATP-dependent DNA ligase. All genes originate from metagenomic DNA that was purified from marine Arctic sediment samples. Candidate genes with sequence homology to proteins of known activity were selected based on tBLAST searches of open reading frames [28] and Pfam domain assignments [30] (Fig. 1, Table 1). Four of the candidates have probable extracellular locations in the native hosts based on the prediction of leader peptides, and three candidates encode two or more cysteine residues in their native polypeptide, some of which could be involved in forming disulfide bonds in the folded structures. The mature proteins have predicted isoelectric points ranging from 4.4 to 9.9, one or two separate domains (for MZ0003 there was no domain prediction), and masses from 32 to 48 kDa. In some cases only portions of the genes were expressed to remove signal peptides, to express only annotated Pfam domains, or to minimize the numbers of low complexity regions and hydrophobic patches in an attempt to improve solubility (Fig. 1). Our candidates are typical representatives of enzyme discovery projects, which cover a set of sequences with diverse properties. Because the DNA was sourced from a permanently cold environment, we presume these genes derive from cold-adapted organisms and wanted to ensure low-temperature expression conditions for the recombinant protein products. The challenge was how to efficiently produce such varied and challenging enzymes in an optimal but universal way, with an acceptable yield and throughput. We decided to use *E. coli* as a host for expression, although this host is known to have lower growth rates and less efficient protein production at low temperatures [38, 39]. To compensate for this, we tested the commercial pCold-II vector [24], and utilized its *cspA* promoter to direct high levels of cold-shock induced expression in *E. coli*. The *cspA* 5′UTR region, which is included in the promoter region of the vector (Fig. 2), adopts a highly stable structure allowing for efficient protein synthesis at low temperatures, such as 15 °C [9, 40–43]. Basal expression from the *cspA* promoter is repressed by a constitutively expressed LacI repressor protein that binds the *lac* operator [24], and a 5′UTR that contains a sequence that traps translation of the recombinant protein and enhances its translation [44–46] (Fig. 2). The pCold-II vector encodes an N-terminal His-tag for purification; so to allow its removal after expression we introduced a TEV protease cleavage site into the multiple cloning site of pCold-II (Fig. 2, Table 2). The genes from our five candidates where successfully inserted into the vector using the RF-cloning method [22, 23, 34].

### The *cspA* promoter gave high total expression and some soluble protein for all candidates

In initial *E. coli* screens, *T*7 promoter-driven expression of the five candidates caused problems with insolubility and toxicity (Additional file 1: Tables S1–S3).

**Fig. 3:**
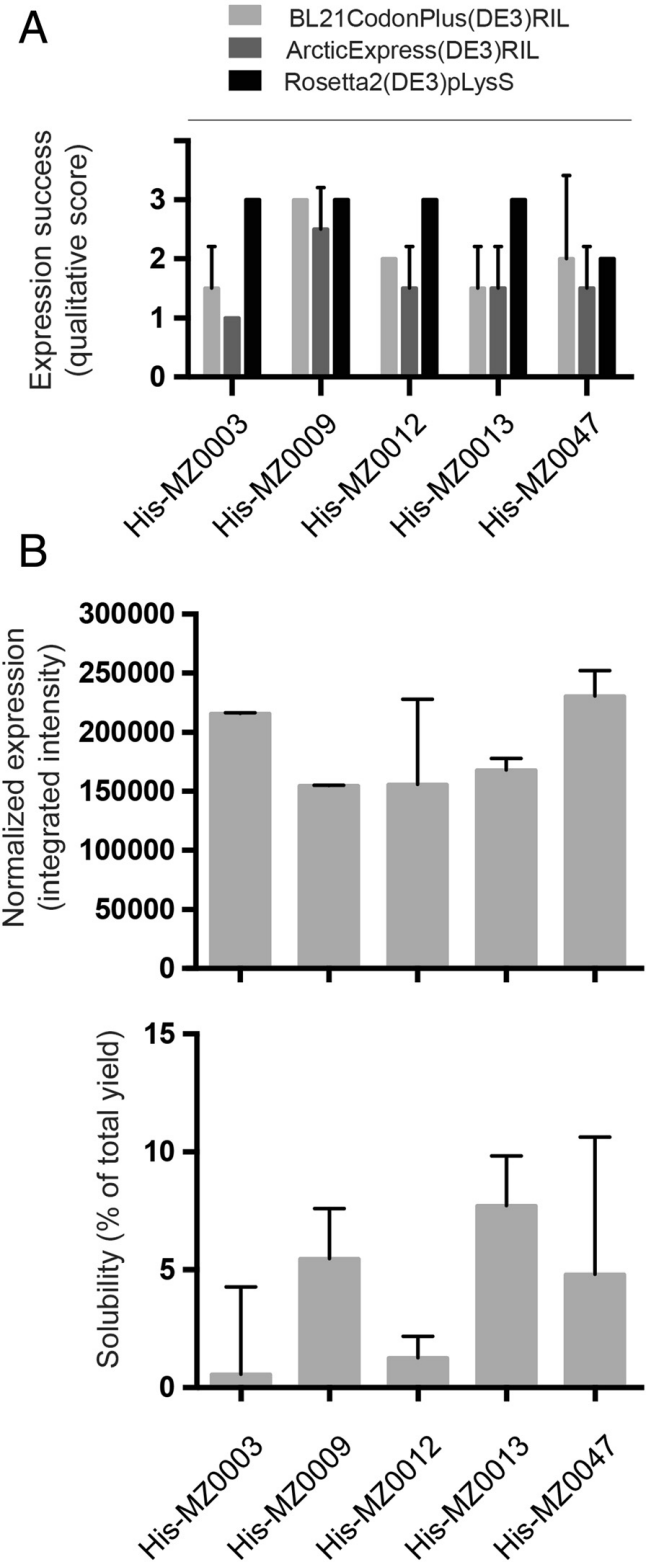
Heterologous expression of candidate genes driven by the *cspA* promoter. **a** *cspA* promoter driven heterologous expression of candidate proteins in three *E. coli* expression strains; BL21CodonPlus(DE3)RIL (*light grey bars*), ArcticExpress(DE3)RIL (*medium grey bars*) and Rosetta2(DE3)pLysS (*black bars*). A qualitative scale for scoring expression success was used as described in the main text. **b** Normalized protein expression (*upper panel*) and solubility (*lower panel*) of candidates after *cspA* driven expression in BL21CodonPlus(DE3)RIL. Error bars show the standard deviation between two independent experiments

To evaluate the performance of the *cspA* promoter for production of our metagenomic proteins, recombinant expression was tested in three common expression strains: BL21CodonPlus(DE3)RIL, ArcticExpress(DE3)RIL and Rosetta2(DE3)pLysS (Fig. 3a). All these strains contain genes encoding rare tRNAs to compensate for the codon usage bias in the Arctic-sourced genes (Table 1) [47–49]. In addition, the strain ArcticExpress(DE3) co-expresses cold-active chaperonines, Cpn60 and Cpn10 from *Oleispira antarctic,* which impart an active protein folding system at low temperatures and is expected to be advantageous for expression of psychrophilic proteins [50]. Successful expression of recombinant proteins, identified from their size on SDS-PAGE, was scored according to the qualitative scale from −1 to 3 described in the Methods section. We found *cspA* drove high expression levels for all candidate proteins in at least one of the strains tested. Interestingly, none of the candidates had negative effects on cell viability, either during transformation, from inoculation to log phase grown or after induction of protein expression (Fig. 3a). Consequently, we were able to express candidate proteins in all strains, including the ArcticExpress(DE3)RIL strain which had previously been unusable in combination with several targets (MZ0003, MZ00013 and MZ0047) under *T*7-driven expression (Tables S1-S3). The explanations for this have not been elucidated; however, our data is consistent with a recent report where combining the *cspA* promoter system with ArcticExpress was successful for expression of cold-adapted proteins [51] and expands the repertoire of expression strains that can be used for these, and most likely other, candidate proteins.

The proportion of recombinant protein in the soluble fraction was evaluated for all candidates expressed under *cspA* in the BL21CodonPlus(DE3)RIL expression strain, according to a semi-quantitative scale. All candidates were expressed in the soluble fraction with a proportion of approximately 10 % or lower compared to the total expression yield (Fig. 3b). Our data is consistent with previous publications reporting that the pCold-vectors can facilitate soluble expression [24, 52, 53] although the extent of solubility depends on the particular protein being expressed.

While the solubility of these candidates expressed in the *cspA* system was not dramatic, this may still be of importance for particular poorly expressed proteins where *T*7 does not appear to be a viable option; as in the cases of MZ0009 and MZ0047 where complete insolubility and toxicity had previously precluded recombinant production of these targets (Additional file 1: Table S1–S3).

### Building a cold-shock inducible vector suite encoding fusion partners

**Fig. 4:**
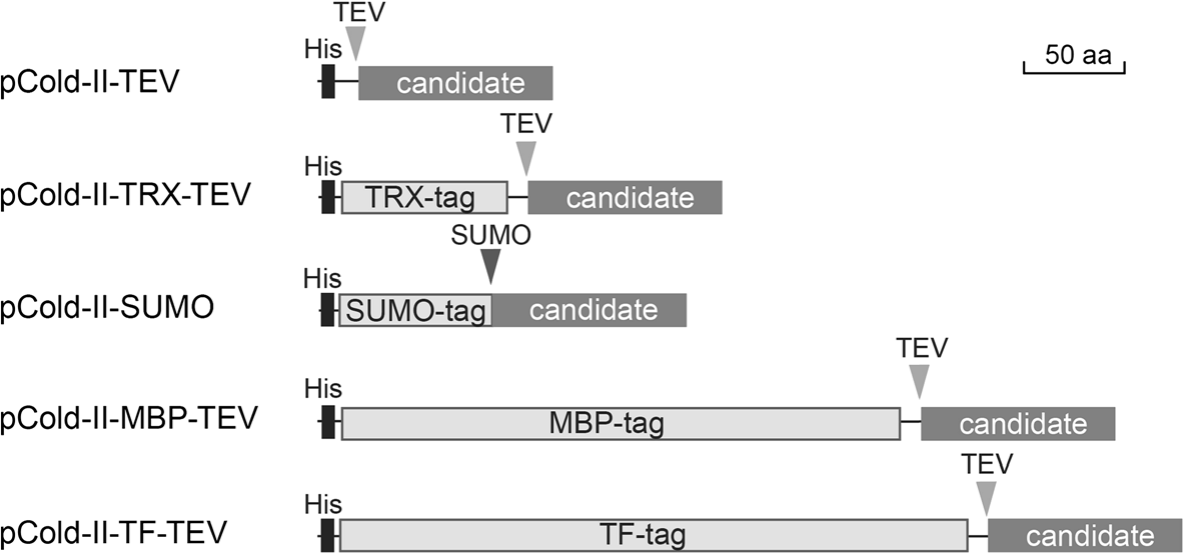
Cartoon of fusion-proteins expressed from the cold-inducible vector suite. Cartoon of the affinity tag (black box) and solubility tags (light grey box) in fusion to candidate sequences (dark grey box) in the recombinant constructs that were generated in this study. Fusion partners are removable in all constructs either by TEV or SUMO protease cleavage; their recognition sites are indicated by triangles. Fusion partners are drawn to scale

As the final yields of soluble protein were still not sufficient for up-scaling for most of our candidates, we were inspired by previous findings [52, 53] to fuse established solubility partners to our recombinant proteins in an attempt to improve their solubility (Fig. 4). We selected the small, soluble protein TRX [16], the large, highly soluble MBP [11–13], and two proteins that have not previously been used in combination with the *cspA* system: the ubiquitin-like SUMO protein [17] and the co-chaperone TF [14, 15], to be used as fusion partners. Previously, these partners have proved successful for soluble expression in large and systematic studies [54–56]. The vector suite was designed to facilitate an efficient and parallel cloning workflow of candidates as fusions to partners. Only three primers per candidate are required to clone each candidate gene into the vector suite: one forward primer to generate the His, His-TRX, His-MBP and His-TF fusion constructs, a second forward primer for the SUMO fusion constructs required to engineer the immediate transition from SUMO-tag to candidate protein, and a reverse primer which was used for all five (Additional file 1: Table S4). Complementing a recent report [57], we demonstrated the application of RF-cloning in building a tailored vector suite and enabling parallel cloning of candidate sequences. All vectors were tested in the *E. coli* Rosetta2(DE3)pLysS strain for soluble expression of fusion partners without candidate genes, and as expected, the majority of recombinant proteins were soluble (data not shown).

### Improvement in solubility with several fusion partners

Small-scale expression of all five candidates from the pCold-II-based vector suite gave significant amounts of recombinant protein, with the exception of the TRX fusion constructs (Table 3). To evaluate the solubility of each candidate expressed from the vector suite, we inspected the SDS-PAGE results of cleared lysates relative to the total yields of recombinant protein produced using the qualitative scores described above. As the His-tag alone does not aid solubility, this construct served as a reference for comparing the improvement in solubility imparted by the fusion partners. We found that the large fusion partners His-MBP and His-TF cloned N-terminal to the candidates gave an outstanding improvement in solubility compared to the His-TRX- and His-SUMO-tags (Table 3). This is in line with previous reports where MBP exceeds smaller tags for soluble expression of both psychrophilic and mesophilic prokaryotic proteins using *T*7-driven [58] and *cspA*-driven expression vectors [53]. Several studies have also been conducted on yeast, plant, mammalian and insect proteins showing a similar performance of MBP [13, 53, 59]. His-TRX-fusion proteins had varying expression levels between independent experiments, and for some constructs (MZ0003, MZ0009 and MZ0047) cell viability was negatively impacted during transformation. Previous findings have shown that the TRX-tag does not work consistently in combination with the pCold system [53]; however as we found that the pCold-II-TRX-TEV vector gave soluble expression of the tag alone, an explanation for the observed toxic effect in our system has not been elucidated.

**Table 3:** Semi-quantitative analysis of fusion construct expression and solubility in Rosetta2(DE3)pLysS^a^

	Target size (kDa)		Mean Expression					Mean Solubility				
Candidate	Native	Recombinant	His	His-SUMO	His-TRX	His-MBP	His-TF	His	His-SUMO	His-TRX	His-MBP	His-TF
MZ0003	48	45.6	3	3	0.5	3	3	1	0.5	1	1.5	3
MZ0009	48	35.8	3	3	−1	3	3	1	1	0	2	3
MZ0012	42	41.6	3	3	2.5	3	3	0.5	0.5	0.5	1.5	3
MZ0013	39	21	3	3	2.5	3	3	1	1.5	1	3	3
MZ0047	32	28.9	2	2.5	1.5	2.5	3	1	0.5	1	2	3

^**a**^evaluated in two independent experiments by a qualitative scoring system described in the text

To summarize, we found that fusion tags, in particular MBP and TF, can be successfully utilized to improve solubility of putatively cold-active enzymes. For all candidates tested in this study, at least one condition was identified which gave rise to sufficient levels of soluble fusion protein to make further attempts at recombinant production feasible.

### Using the vector suite, a functional chitinase was produced

**Fig. 5:**
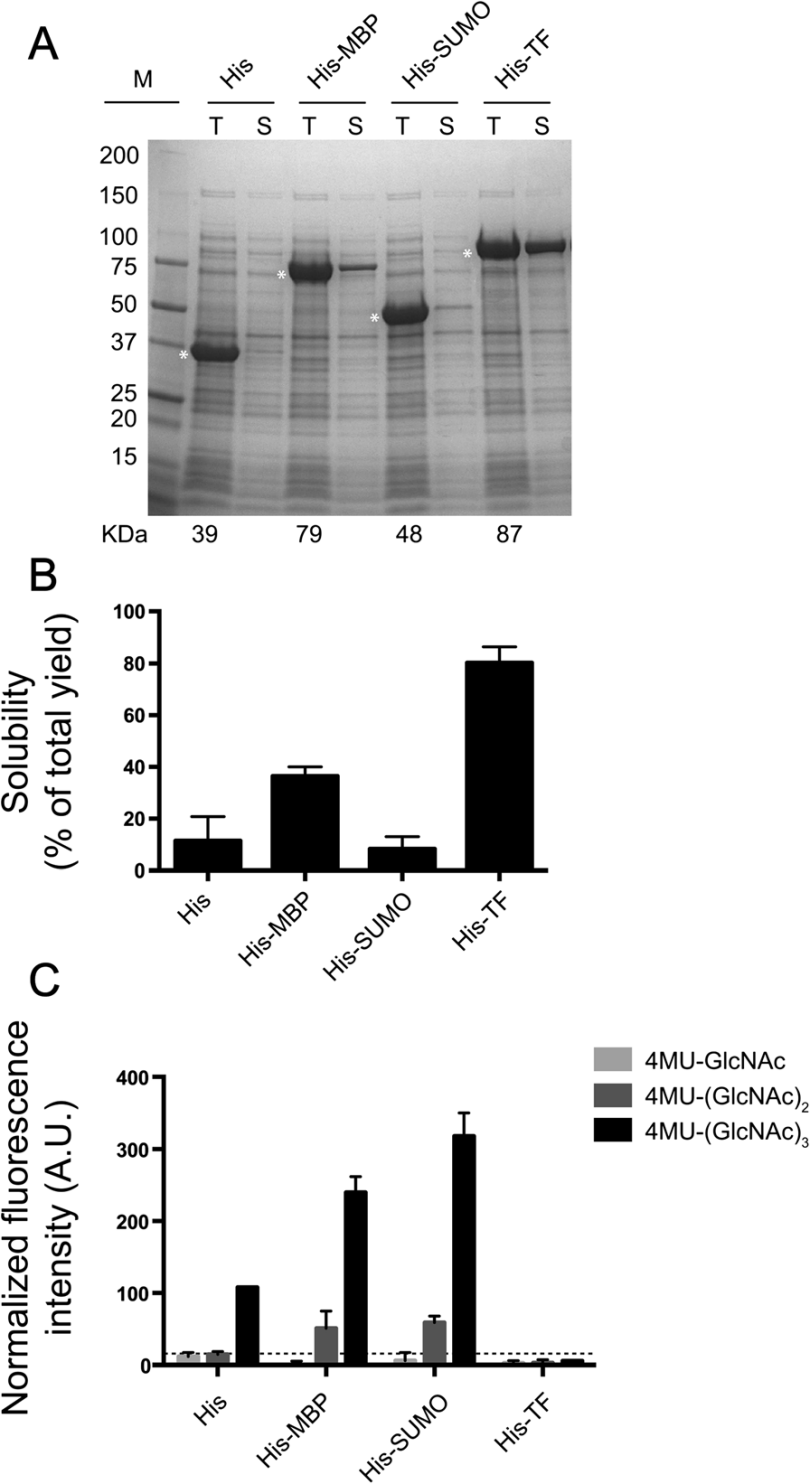
Comparison of expression level, solubility and activity of the cold-shock inducible, recombinant chitinase. **a** A representative SDS-PAGE gel showing the total protein produced (T) and the soluble fraction (S) in lysed Rosetta2(DE3)pLysS extracts of MZ0009 expressed from the pCold-vector suite. Asterisks indicate the presence of recombinant proteins of theoretical expected mass (given below figure). M, Protein standard in kilodaltons (kDa). **b** Semi-quantitative calculation of soluble fraction of the MZ0009 fusion proteins. Error bars show variation in two independent experiments. **c** Chitinolytic activity, presented as normalized fluorescence intensities, towards three synthetic 4-methylumbelliferone-labelled (4MU) chitin analogs in the cleared lysates containing MZ0009 fused to fusion proteins. Background activity from expression of ‘empty’ vectors is indicated with a dashed baseline. A.U., arbitrary units*.* Error bars show variation in two replicates in one representative experiment

As a proof of principle, the candidate MZ0009 was chosen for functional investigation. This candidate showed a marked improvement in expression under the *cspA* promoter in Rosetta2(DE3)pLysS; previous *T*7-based experiments resulted in completely insoluble protein (Tables S1-S3), while *cspA*-controlled expression produced 5-10 % soluble product with only a His-tag (Fig. 3b), which increased to 80-100 % when His-MBP- and His-TF-tags were used (Table 3). Semi-quantitative evaluation of MZ0009 solubility with different tags showed an improvement in the order: TF > MBP > SUMO = His (Fig. 5a and b). The two latter tags show considerable variation in solubility between parallel experiments, and were therefore considered equal. As described above, no transformants were obtained when MZ0009 was cloned in fusion with the His-TRX-tag.

Based on amino acid sequence similarity, the MZ0009 candidate was annotated as a chitinase belonging to the glycoside hydrolase family 18 (GH18) [60, 61]. This class of enzymes hydrolyze the glycosidic β-1,4-linkage between *N-*acetylglucosamine (GlcNAc) units of the chitin biopolymer, and can be endo- and exo*-*acting [62–65]. A putatively cold-adapted chitinase of the GH18-family is industrially relevant for treatment of chitin-rich biomass at low temperatures, for biocontrol of phytopathogens in cold environments and prevention of microbial spoilage of refrigerated food [64, 66–68].

The conserved catalytic DxDxE motif [69, 70] was found in MZ0009 based on an alignment of the MZ0009 sequence with characterized GH18 chitinases (Additional file 2: Figure S1). The motif includes the essential, catalytic residues asp147, asp149 and glu151, corresponding to asp140, asp142 and glu144 respectively in the well-characterized ChiB from *Serratia marcescens* [71–73]. To confirm this sequence annotation, clarified lysates containing MZ0009 fused to each of the four tags were assayed for chitinase activity (Fig. 5c) using three synthetic 4-methylumbelliferone (4MU)-labeled chitin analogs. The His-, His-MBP- and His-SUMO-tagged fusion-constructs of MZ0009 displayed pronounced chitinolytic activity on the 4MU-β-D-*N*,*N*’,*N*”-triacetylchitotriose (4MU-(GlcNAc)_3_) substrate (Fig. 5c), while lower levels of chitinase activity were detected on the 4MU-*N*,*N*’-diacetyl-β-D-chitobioside (4MU-(GlcNAc)_2_) substrate with the His-SUMO and His-MBP constructs (Fig. 5c). As exo-chitinolytic enzymes act on terminal *N*-acetylglucosamine residues to remove either monosaccharides or disaccharides, endochitinases are required to release fluorescent products from 4MU-(GlcNAc)_3_ [74], indicating that MZ0009 is an endochitinase. Interestingly the His-SUMO-fusion construct, which gave little or no improvement in solubility compared to the His-only construct (Fig. 5a and b), showed the most pronounced chitinolytic activity (Fig. 5c). This indicates that soluble expression, which was equivalent for both constructs, does not necessarily equate to a functional enzyme, as the His-tagged control was significantly less active than the His-SUMO fusion. As none of the solubility tags had chitinolytic activity when expressed alone (data not shown) this improvement in activity can only be ascribed to an influence of the fusion partner on MZ0009 folding. Our data strengthens the observation that MBP and SUMO partners can be used to promote correct folding of difficult-to-express proteins [58, 75]. In addition to confirming the successful folding of the MZ0009 fusion constructs, we have provided a preliminary characterization of an endo-chitinase. As a proof of concept, a functional chitinase, representing the first recombinant endo-chitinase from an environmental Arctic source was produced by using the cold-shock inducible vector suite.

Although approximately 80 % of the His-TF fusion construct of MZ0009 was expressed as a soluble protein, this fusion construct has no significant activity towards 4MU-(GlcNAc)_3_ in our chitinolytic assay (Fig. 5c). We attempted to remove the His-TF-tag utilizing the TEV-protease site between the tag and candidate (Fig. 2), but TEV protease treatment in a cleared lysate resulted in only approximately 10 % cleavage of the fusion protein (data not shown), which may indicate that the TEV-site in the linker (Table 2) was inaccessible to the TEV protease. A known drawback of fusion tags is they may interfere with the activity of the candidate protein by sterically hindering access of substrate to the catalytic site, or by causing the candidate to adopt a non-functional, though soluble, conformation [76, 77]. Although this property can be successfully exploited for expression of toxic targets [78], it is generally undesirable and generates false-positive results as the candidate may remain inactive upon removal of the tag. The importance of the length and nature of the linker joining the candidate to the fusion partner has been recognized and discussed in several studies [79–82], however a systematic study of optimal linker length has not been carried out for TF-fusion constructs and will be required to fully exploit its potential as a solubility and co-folding partner in recombinant protein expression.

## Conclusions

Our aim was to develop an efficient screening system for putatively cold-active enzymes intended for biotechnological purposes, using metagenomic DNA from the marine Arctic environment as a source of candidate genes. To facilitate this, a vector suite based on the *cspA* promoter was designed for fast-cloning and low-temperature heterologous expression. Our data show that the *cspA* promoter can be utilized for low-temperature production of high levels of expression for putatively cold-adapted candidate genes. The *cspA* system also mitigated the toxic effects that were observed with several candidates under *T*7-driven expression. We found that combining the candidate genes with the established MBP fusion partner substantially improved the solubility of the recombinant proteins. We have also extended the utility of the SUMO protein and TF fusion proteins for soluble protein production under *cspA* promoter control. In workflows typical of an enzyme discovery project, a robust production procedure to obtain sufficient quantities of active enzyme is typically part of the initial screening phase. From this perspective, parallel testing of different fusion tags is extremely useful as it can help improve the efficiency of the production procedure. In summary, the vector suite facilitates a low-temperature optimized system for heterologous expression of putatively cold-active enzymes in fusion to both small and large fusion partners. The process of generating all tag-candidate combinations is simplified by using our parallel cloning strategy for candidate gene insertion.

As a proof of concept, we showed that the Arctic-sourced MZ0009 candidate, a putative GH18 family member, was expressed in a soluble and functional form in the optimized expression system. Recombinant MZ0009 showed activity towards synthetic chitin analogs when cell lysates with overexpressed fusion proteins were tested in an activity assay. The highest activity was found for the 4MU-(GlcNAc)_3_ substrate, indicating that the MZ0009 protein is an endo-chitinase.

We envision this expression system being employed in further bioprospecting endeavors for psychrophilic proteins, and suggest that it can provide a good starting point for enzyme discovery and development. Moreover, the inclusion of His-tag and either TEV or SUMO protease cleavage sites in all constructs would allow large-scale purification and tag removal to proceed directly from successful screens for crystallization studies. Finally, we suggest that such a cold-shock inducible system could be advantageous for the heterologous expression of toxic mesophilic and thermophilic proteins, where properties of the proteins are deleterious to the host cell growth [83, 84]. If such proteins were expressed at temperatures below their activity optima, we would expect a decrease in their toxicity during recombinant production [85].

## Additional files

Additional file 1:
**Description of dataset: Initial** ***T*** **7 expression data in Table S1, S2 and S3.** Primer information in **Table S4**. (DOCX 41 kb) Additional file 2:
**Sequence alignment of MZ0009 with characterized GH18 chitinases.** (JPEG 3038 kb).

## Abbreviations

GlcNAc: N-acetylglucosamine
*c**spA* promoter: Cold shock protein A promoter
IPTG: β–thiogalactopyranoside
His: Hexahistidine
TRX: Thioredoxin
SUMO: Small ubiquitin-like modifier
TF: Trigger factor
MBP: Maltose-binding protein
TEE: Translation-enhancing element
RBS: Ribosome binding site
4MU: 4-methylumbelliferone
DW24: 24 deep well plate
RF: Restriction-free
TEV: Tobacco etch virus
GH18: Glycoside hydrolase family 18
Cpn10/60: Chaperonine 10/60

## References

[1] Demirjian DC, Moris-Varas F, Cassidy CS (2001). Enzymes from extremophiles. Curr Opin Chem Biol.

[2] van den Burg B (2003). Extremophiles as a source for novel enzymes. Curr Opin Microbiol.

[3] Ferrer M, Golyshina O, Beloqui A, Golyshin PN (2007). Mining enzymes from extreme environments. Curr Opin Microbiol.

[4] Feller G (2013). Psychrophilic enzymes: from folding to function and biotechnology. Scientifica.

[5] Feller G, Le Bussy O, Gerday C (1998). Expression of psychrophilic genes in mesophilic hosts: assessment of the folding state of a recombinant alpha-amylase. Appl Environ Microbiol.

[6] Smalas AO, Leiros HK, Os V, Willassen NP (2000). Cold adapted enzymes. Biotechnol Annu Rev.

[7] Feller G (2007). Life at low temperatures: is disorder the driving force?. Extremophiles.

[8] Gerday C, Aittaleb M, Bentahir M, Chessa J-P, Claverie P, Collins T (2000). Cold-adapted enzymes: from fundamentals to biotechnology. Trends Biotechnol.

[9] Tanabe H, Goldstein J, Yang M, Inouye M (1992). Identification of the promoter region of the Escherichia coli major cold shock gene, cspA. J Bacteriol.

[10] Vasina JA, Baneyx F (1996). Recombinant protein expression at low temperatures under the transcriptional control of the major Escherichia coli cold shock promoter cspA. Appl Environ Microbiol.

[11] Bedouelle H, Duplay P (1988). Production in Escherichia coli and one-step purification of bifunctional hybrid proteins which bind maltose. Export of the Klenow polymerase into the periplasmic space. Eur Jo Biochem.

[12] di Guan C, Li P, Riggs PD, Inouye H (1988). Vectors that facilitate the expression and purification of foreign peptides in Escherichia coli by fusion to maltose-binding protein. Gene.

[13] Kapust RB, Waugh DS (1999). Escherichia coli maltose-binding protein is uncommonly effective at promoting the solubility of polypeptides to which it is fused. Protein Sci.

[14] Esaki K, Terashima Y, Toda E, Yoshinaga S, Araki N, Matsushima K (2011). Expression and purification of human FROUNT, a common cytosolic regulator of CCR2 and CCR5. Protein Expr Purif.

[15] Hoffmann A, Merz F, Rutkowska A, Zachmann-Brand B, Deuerling E, Bukau B (2006). Trigger factor forms a protective shield for nascent polypeptides at the ribosome. J Biol Chem.

[16] LaVallie ER, DiBlasio EA, Kovacic S, Grant KL, Schendel PF, McCoy JM (1993). A thioredoxin gene fusion expression system that circumvents inclusion body formation in the E. coli cytoplasm. Biotechnology (Nature Publishing Company).

[17] Malakhov MP, Mattern MR, Malakhova OA, Drinker M, Weeks SD, Butt TR (2004). SUMO fusions and SUMO-specific protease for efficient expression and purification of proteins. J Struct Funct Genomics.

[18] Smith MC, Furman TC, Ingolia TD, Pidgeon C (1988). Chelating peptide-immobilized metal ion affinity chromatography. A new concept in affinity chromatography for recombinant proteins. J Biol Chem.

[19] Hochuli E (1988). Large-scale chromatography of recombinant proteins. J Chromatogr.

[20] CSteen J, Uhlen M, Hober S, Ottosson J. High-throughput protein purification using an automated set-up for high-yield affinity chromatography. Protein Expr Purif. 2006;46(2):173–8. 10.1016/j.pep.2005.12.010 16483795

[21] Schafer F, Romer U, Emmerlich M, Blumer J, Lubenow H, Steinert K (2002). Automated high-throughput purification of 6xHis-tagged proteins. J Biomolec Tech.

[22] van den Ent F, Lowe J (2006). RF cloning: a restriction-free method for inserting target genes into plasmids. J Biochem Biophys Methods.

[23] Unger T, Jacobovitch Y, Dantes A, Bernheim R, Peleg Y (2010). Applications of the Restriction Free (RF) cloning procedure for molecular manipulations and protein expression. J Struct Biol.

[24] Qing G, Ma LC, Khorchid A, Swapna GV, Mal TK, Takayama MM (2004). Cold-shock induced high-yield protein production in Escherichia coli. Nat Biotechnol.

[25] Fu J, Leiros HK, de Pascale D, Johnson KA, Blencke HM, Landfald B (2013). Functional and structural studies of a novel cold-adapted esterase from an Arctic intertidal metagenomic library. Appl Microbiol Biotechnol.

[26] Margulies M, Egholm M, Altman WE, Attiya S, Bader JS, Bemben LA (2005). Genome sequencing in microfabricated high-density picolitre reactors. Nature.

[27] Zhu W, Lomsadze A, Borodovsky M (2010). Ab initio gene identification in metagenomic sequences. Nucleic Acids Res.

[28] Altschul SF, Gish W, Miller W, Myers EW, Lipman DJ (1990). Basic local alignment search tool. J Mol Biol.

[29] Petersen TN, Brunak S, von Heijne G, Nielsen H (2011). SignalP 4.0: discriminating signal peptides from transmembrane regions. Nat Methods.

[30] Finn RD, Bateman A, Clements J, Coggill P, Eberhardt RY, Eddy SR (2014). Pfam: the protein families database. Nucleic Acids Res.

[31] Arnold K, Bordoli L, Kopp J, Schwede T (2006). The SWISS-MODEL workspace: a web-based environment for protein structure homology modelling. Bioinformatics (Oxford, England).

[32] Wilkins MR, Gasteiger E, Bairoch A, Sanchez JC, Williams KL, Appel RD (1999). Protein identification and analysis tools in the ExPASy server. Methods Mole Biol (Clifton, NJ).

[33] Gräslund S, Sagemark J, Berglund H, Dahlgren L-G, Flores A, Hammarström M (2008). The use of systematic N- and C-terminal deletions to promote production and structural studies of recombinant proteins. Protein Expr Purif.

[34] Lund BA, Leiros HK, Bjerga GE (2014). A high-throughput, restriction-free cloning and screening strategy based on ccdB-gene replacement. Microb Cell Fact.

[35] Bond SR, Naus CC (2012). RF-Cloning.org: an online tool for the design of restriction-free cloning projects. Nucleic Acids Res.

[36] Busso D, Delagoutte-Busso B, Moras D (2005). Construction of a set Gateway-based destination vectors for high-throughput cloning and expression screening in Escherichia coli. Anal Biochem.

[37] Vincentelli R, Cimino A, Geerlof A, Kubo A, Satou Y, Cambillau C (2011). High-throughput protein expression screening and purification in Escherichia coli. Methods.

[38] Broeze RJ, Solomon CJ, Pope DH (1978). Effects of low temperature on in vivo and in vitro protein synthesis in Escherichia coli and Pseudomonas fluorescens. J Bacteriol.

[39] Shaw MK, Ingraham JL (1967). Synthesis of macromolecules by Escherichia coli near the minimal temperature for growth. J Bacteriol.

[40] Goldenberg D, Azar I, Oppenheim AB (1996). Differential mRNA stability of the cspA gene in the cold-shock response of Escherichia coli. Mol Microbiol.

[41] Brandi A, Pietroni P, Gualerzi CO, Pon CL (1996). Post-transcriptional regulation of CspA expression in Escherichia coli. Mol Microbiol.

[42] Mitta M, Fang L, Inouye M (1997). Deletion analysis of cspA of Escherichia coli: requirement of the AT-rich UP element for cspA transcription and the downstream box in the coding region for its cold shock induction. Mol Microbiol.

[43] Fang L, Jiang W, Bae W, Inouye M (1997). Promoter-independent cold-shock induction of cspA and its derepression at 37 degrees C by mRNA stabilization. Mol Microbiol.

[44] Goldstein J, Pollitt NS, Inouye M (1990). Major cold shock protein of Escherichia coli. Proc Natl Acad Sci U S A.

[45] Xia B, Etchegaray JP, Inouye M (2001). Nonsense mutations in cspA cause ribosome trapping leading to complete growth inhibition and cell death at low temperature in Escherichia coli. J Biol Chem.

[46] Jiang W, Fang L, Inouye M (1996). Complete growth inhibition of Escherichia coli by ribosome trapping with truncated cspA mRNA at low temperature. Genes Cells.

[47] Rosano GL, Ceccarelli EA (2009). Rare codon content affects the solubility of recombinant proteins in a codon bias-adjusted Escherichia coli strain. Microb Cell Fact.

[48] Goldman E, Rosenberg AH, Zubay G, Studier FW (1995). Consecutive low-usage leucine codons block translation only when near the 5′ end of a message in Escherichia coli. J Mol Biol.

[49] Kane JF (1995). Effects of rare codon clusters on high-level expression of heterologous proteins in Escherichia coli. Curr Opin Biotechnol.

[50] Ferrer M, Chernikova TN, Yakimov MM, Golyshin PN, Timmis KN (2003). Chaperonins govern growth of Escherichia coli at low temperatures. Nat Biotechnol.

[51] Ueda M, Ito A, Nakazawa M, Miyatake K, Sakaguchi M, Inouye K (2014). Cloning and expression of the cold-adapted endo-1,4-beta-glucanase gene from Eisenia fetida. Carbohydr Polym.

[52] Hayashi K, Kojima C (2008). pCold-GST vector: a novel cold-shock vector containing GST tag for soluble protein production. Protein Expr Purif.

[53] Hayashi K, Kojima C (2010). Efficient protein production method for NMR using soluble protein tags with cold shock expression vector. J Biomol NMR.

[54] Bird LE (2011). High throughput construction and small scale expression screening of multi-tag vectors in Escherichia coli. Methods.

[55] Marblestone JG, Edavettal SC, Lim Y, Lim P, Zuo X, Butt TR (2006). Comparison of SUMO fusion technology with traditional gene fusion systems: enhanced expression and solubility with SUMO. Protein Sci.

[56] Hammarstrom M, Hellgren N, van Den Berg S, Berglund H, Hard T (2002). Rapid screening for improved solubility of small human proteins produced as fusion proteins in Escherichia coli. Protein Sci.

[57] Correa A, Ortega C, Obal G, Alzari P, Vincentelli R, Oppezzo P (2014). Generation of a vector suite for protein solubility screening. Front Microbiol.

[58] Niiranen L, Espelid S, Karlsen CR, Mustonen M, Paulsen SM, Heikinheimo P (2007). Comparative expression study to increase the solubility of cold adapted Vibrio proteins in Escherichia coli. Protein Expr Purif.

[59] Shih YP, Kung WM, Chen JC, Yeh CH, Wang AH, Wang TF (2002). High-throughput screening of soluble recombinant proteins. Protein Sci.

[60] Lombard V, Golaconda Ramulu H, Drula E, Coutinho PM, Henrissat B (2014). The carbohydrate-active enzymes database (CAZy) in 2013. Nucleic Acids Res.

[61] Henrissat B, Davies G (1997). Structural and sequence-based classification of glycoside hydrolases. Curr Opin Struct Biol.

[62] Howard MB, Ekborg NA, Taylor LE, Weiner RM, Hutcheson SW (2004). Chitinase B of “Microbulbifer degradans” 2–40 contains two catalytic domains with different chitinolytic activities. J Bacteriol.

[63] Horn SJ, Sørlie M, Vaaje-Kolstad G, Norberg AL, Synstad B, Vårum KM (2006). Comparative studies of chitinases A, B and C from Serratia marcescens. Biocatal Biotransform.

[64] Lonhienne T, Mavromatis K, Vorgias CE, Buchon L, Gerday C, Bouriotis V (2001). Cloning, sequences, and characterization of two chitinase genes from the Antarctic Arthrobacter sp. strain TAD20: isolation and partial characterization of the enzymes. J Bacteriol.

[65] Hult EL, Katouno F, Uchiyama T, Watanabe T, Sugiyama J (2005). Molecular directionality in crystalline beta-chitin: hydrolysis by chitinases A and B from Serratia marcescens 2170. Biochem J.

[66] Goni O, Sanchez-Ballesta MT, Merodio C, Escribano MI (2013). Two cold-induced family 19 glycosyl hydrolases from cherimoya (Annona cherimola) fruit: an antifungal chitinase and a cold-adapted chitinase. Phytochemistry.

[67] Ramli AN, Mahadi NM, Rabu A, Murad AM, Bakar FD, Illias RM (2011). Molecular cloning, expression and biochemical characterisation of a cold-adapted novel recombinant chitinase from Glaciozyma antarctica PI12. Microb Cell Fact.

[68] Orikoshi H, Baba N, Nakayama S, Kashu H, Miyamoto K, Yasuda M (2003). Molecular analysis of the gene encoding a novel cold-adapted chitinase (ChiB) from a marine bacterium, Alteromonas sp. strain O-7. J Bacteriol.

[69] Watanabe T, Kobori K, Miyashita K, Fujii T, Sakai H, Uchida M (1993). Identification of glutamic acid 204 and aspartic acid 200 in chitinase A1 of Bacillus circulans WL-12 as essential residues for chitinase activity. J Biol Chem.

[70] van Aalten DM, Komander D, Synstad B, Gaseidnes S, Peter MG, Eijsink VG (2001). Structural insights into the catalytic mechanism of a family 18 exo-chitinase. Proc Natl Acad Sci U S A.

[71] Vaaje-Kolstad G, Houston DR, Rao FV, Peter MG, Synstad B, van Aalten DM (2004). Structure of the D142N mutant of the family 18 chitinase ChiB from Serratia marcescens and its complex with allosamidin. Biochim Biophys Acta.

[72] Kolstad G, Synstad B, Eijsink VG, van Aalten DM (2002). Structure of the D140N mutant of chitinase B from Serratia marcescens at 1.45 A resolution. Acta Crystallogr D Biol Crystallogr.

[73] Synstad B, Gaseidnes S, Van Aalten DM, Vriend G, Nielsen JE, Eijsink VG (2004). Mutational and computational analysis of the role of conserved residues in the active site of a family 18 chitinase. Eur J Biochem.

[74] Tronsmo A, Harman GE (1993). Detection and quantification of N-acetyl-beta-D-glucosaminidase, chitobiosidase, and endochitinase in solutions and on gels. Anal Biochem.

[75] Butt TR, Edavettal SC, Hall JP, Mattern MR (2005). SUMO fusion technology for difficult-to-express proteins. Protein Expr Purif.

[76] Nallamsetty S, Waugh DS (2006). Solubility-enhancing proteins MBP and NusA play a passive role in the folding of their fusion partners. Protein Expr Purif.

[77] Chant A, Kraemer-Pecore CM, Watkin R, Kneale GG (2005). Attachment of a histidine tag to the minimal zinc finger protein of the Aspergillus nidulans gene regulatory protein AreA causes a conformational change at the DNA-binding site. Protein Expr Purif.

[78] Williamson A, Pedersen H (2014). Recombinant expression and purification of an ATP-dependent DNA ligase from Aliivibrio salmonicida. Protein Expr Purif.

[79] Chen X, Zaro JL, Shen W-C (2013). Fusion protein linkers: Property, design and functionality. Adv Drug Deliv Rev.

[80] Gromek KA, Meddaugh HR, Wrobel RL, Suchy FP, Bingman CA, Primm JG (2013). Improved expression and purification of sigma 1 receptor fused to maltose binding protein by alteration of linker sequence. Protein Expr Purif.

[81] Smyth DR, Mrozkiewicz MK, McGrath WJ, Listwan P, Kobe B (2003). Crystal structures of fusion proteins with large-affinity tags. Protein Sci.

[82] Arai R, Wriggers W, Nishikawa Y, Nagamune T, Fujisawa T (2004). Conformations of variably linked chimeric proteins evaluated by synchrotron X-ray small-angle scattering. Proteins.

[83] Tamura M, Ito K, Kunihiro S, Yamasaki C, Haragauchi M (2011). Production of human beta-actin and a mutant using a bacterial expression system with a cold shock vector. Protein Expr Purif.

[84] Kim EK, Moon JC, Lee JM, Jeong MS, Oh C, Ahn SM (2012). Large-scale production of soluble recombinant amyloid-beta peptide 1–42 using cold-inducible expression system. Protein Expr Purif.

[85] Mujacic M, Cooper KW, Baneyx F (1999). Cold-inducible cloning vectors for low-temperature protein expression in Escherichia coli: application to the production of a toxic and proteolytically sensitive fusion protein. Gene.

