# Supplementary material for: Cold shock induction of recombinant Arctic environmental genes
Source: BMC Biotechnol. 2015 Aug 19;15:78. doi: 10.1186/s12896-015-0185-1 (PMC4544801; doi:10.1186/s12896-015-0185-1)
Supplement: Additional file 1: — Description of dataset: Initial T 7 expression data in Table S1, S2 and S3. Primer information in Table S4. (DOCX 41 kb) [file 12896_2015_185_MOESM1_ESM.docx]

Supplementary material

**Cold shock induction of recombinantly-expressed Arctic environmental genes**

*Gro Elin Kjæreng Bjerga*^a, 1^*, Adele Kim Williamson*^a^

^a^Norstruct, Department of Chemistry, Faculty of Science and Technology, University of Tromsø, N-9037 Tromsø, Norway

^1^Present address: Uni Research AS, Centre for Applied Biotechnology, Thormøhlensgt. 55, N-5008 Bergen, Norway

**Corresponding author:**

Gro Elin Kjæreng Bjerga

Uni Research AS, Centre for Applied Biotechnology

Thormøhlensgt. 55

N-5008 Bergen, Norway

**Table S1.** Constructs cloned under control of the T7 promoter

| **Target** | **Construct** | **Plasmid** | **Truncation** | **Leader sequence** | **Fusion tag** |
| --- | --- | --- | --- | --- | --- |
| MZ0003 | 1 | pET-DEST42 | No | Native | His (C) |
|  | 2 | pET26b(+) | N-terminal (1-25) | PelB | His (C) |
|  | 3 | pET32 | N-terminal (1-25) | No | TRX-His (N), TEV |
|  | 4 | pET30 | N-terminal (1-25) | No | His (N), TEV |
|  | 5 | pET30 | N-terminal (1-25) | No | His (C) |
| MZ0009 | 1 | pET-DEST42 | None | Native | His (C) |
|  | 2 | pDEST17 | N-terminal (1-26) | No | His (N), TEV |
|  | 3 | pDEST17 | N-terminal (1-26)  C-terminal (359-449) | No | His (N), TEV |
|  | 4 | pDEST17 | N-terminal (1-26)  C-terminal (340-449) | No | His (N), TEV |
|  | 5 | pDEST14 | No | Native | None |
| MZ0012 | 1 | pET-DEST42 | None | No | His (C) |
|  | 2 | pDEST17 | No | No | His (N), TEV |
| MZ0013 | 1 | pET-DEST42 | None | Native | His (C) |
|  | 2 | pDEST17 | N-terminal (1-38)  C-terminal (229-352) | No | His (N), TEV |
| MZ0047 | 1 | pDEST15 | N-terminal (1-33) | No | GST (N), TEV |
|  | 2 | pET-DEST42 | No | Native | His (C) |
|  | 3 | pET-DEST42 | N-terminal (1-33) | No | His (C) |
|  | 4 | pET26b(+) | N-terminal (1-33) | PelB | His (C) |
|  | 5 | pET26b(+)^a^ | No | Native | His (C) |
|  | 6 | pET26b(+)^a^ | N-terminal (1-33) | No | His (C) |
|  | 7 | pDEST17 | N-terminal (1-33) | No | His (N), TEV |

a) using sites that remove the *E. coli* PelB leader sequences encoded in the vector

**Table S2.** Expression conditions tested from T7 constructs. ++ indicates strong expression, + indicates a visible band, - indicates no expression T indicates toxicity (no cell growth).

| **Target** | **Construct** | **10-12 (ON)** | **15 (ON)** | **20 (ON)** | **37 (ON)** | **Comment** |
| --- | --- | --- | --- | --- | --- | --- |
| MZ0003 | 1 |  | - ^a,^ |  | + ^a^ |  |
|  | 2 |  | + ^a^ |  | ++ ^a^ | No over expression in the periplasmic fraction |
|  | 3 |  | + ^a, d^ | +^, c^ | ++ ^a^ |  |
|  | 4 |  | +^b, c^ T^d^ | +^a^ |  |  |
|  | 5 | + ^d^ |  |  |  |  |
| MZ0009 | 1 |  |  | -^b^ | -^b^ |  |
|  | 2 |  |  | ++^c^ | ++^c^ |  |
|  | 3 |  | ++ ^b, c, d^ | ++^c^ | ++^c^ |  |
|  | 4 |  |  | ++^c^ | ++^c^ |  |
|  | 5 |  |  | +^c^ | +^c^ |  |
| MZ0012 | 1 |  |  | -^a,b^ | -^a,b^ |  |
|  | 2 | + ^c^ | ++ ^b, c,^ +^d^ |  |  |  |
| MZ0013 | 1 |  |  | -^a,^ +^b^ | -^a,^+^b^ |  |
|  | 2 | + ^c^ | ++ ^c^ +^b^ T^d^ |  |  |  |
| MZ0047 | 1 |  | ++ ^a^ |  | ++ ^a^ | Batch variation in expression |
|  | 2 |  | T ^a^ |  | T ^a^ |  |
|  | 3 |  | T ^a^ |  | T ^a^ |  |
|  | 4 |  | + ^a^ | + ^a^ | ++ ^a^ | No over expression in the periplasmic fraction. Mixture of processed and unprocessed leader peptide |
|  | 5 |  |  | T ^a^ |  |  |
|  | 6 |  |  | T ^a^ |  |  |
|  | 7 |  | + ^c^ T ^b, d^ |  |  |  |

1. BL21Star pLys pRare
2. BL21CodonPlus(DE3)RIL
3. Rosetta2(DE3)pLysS
4. ArcticExpress(DE3)RIL

**Table S3.** Solubility of proteins expressed under T7. S = soluble, (S)= small fraction soluble but most in the pellet, I = all insoluble, - is no expression, T is toxicity.

| **Target** | **Construct** | **10-12(ON)** | **15 (ON)** | **20 (ON)** | **37 (ON)** | **Comment** |
| --- | --- | --- | --- | --- | --- | --- |
| MZ0003 | 1 |  | - ^a^ |  | I ^a^ |  |
|  | 2 |  | (S) ^a^ |  | I ^a^ | Soluble intracellular protein only indicating the leader peptide had not been processed |
|  | 3 |  | (S) ^a, d^ | (S) ^c^ | I ^a^ | Batch variation in solubility |
|  | 4 |  | (S)^b^ | (S)^a^ |  |  |
|  | 5 | I ^d^ |  |  |  |  |
| MZ0009 | 1 |  |  | -^b^ | -^b^ |  |
|  | 2 |  |  | (S)^c^ | I^c^,(S)^c^ |  |
|  | 3 |  | (S)^b,d^I^c^ |  | I^c^ |  |
|  | 4 |  |  |  | I^c^ |  |
| MZ0012 | 1 |  |  | -^a,b^ | -^a,b^ |  |
|  | 2 | I^c^ | I^c^, (S)^b, d^ |  |  |  |
| MZ0013 | 1 |  |  | -^a^ | -^a^ |  |
|  | 2 | I^c^ | I^c^, (S)^b^,T^d^ |  |  |  |
| MZ0047 | 1 |  | (S) ^a^ |  | I ^a^ |  |
|  | 2 |  | T ^a^ |  | T ^a^ |  |
|  | 3 |  | T ^a^ |  | T ^a^ |  |
|  | 4 |  | (s) ^a^ | (s) ^a^ | I ^a^ | Mixture of processed and unprocessed leader peptide |
|  | 5 |  |  | T ^a^ |  |  |
|  | 6 |  |  | T ^a^ |  |  |
|  | 7 |  | T ^b^ |  |  |  |

1. BL21Star pLysS pRARE
2. BL21CodonPlus(DE3)RIL
3. Rosetta2(DE3)pLysS
4. ArcticExpress(DE3)RIL

**Table S4. Primers used for generation of vectors and amplification and sequencing of cloned constructs.**

| **Primer name (restriction site)** | **Sequence^a^ (5'-3')** | **Purpose** |
| --- | --- | --- |
| TEV5' (BamHI) | GATCCGAGAACCTTTACTTCCAGGGGG | Primers for TEV insertion to pCold-II by oligonucleotide cloning |
| TEV3' (BamHI) | GATCCCCCCTGGAAGTAAAGGTTCTCG | Primers for TEV insertion to pCold-II by oligonucleotide cloning |
| pCold-SUMO-F | CCATGAATCACAAAGTGCATCATCATCATCATCATAGCGACTCGGAAGTGAAC | Primers for gene-specific amplification (1^st^ PCR) designed for RF cloning of pCold-II-SUMO |
| pCold-SUMO-R | GGTCGACAAGCTTGAATTCGGATCCACCGCCGATTTGTTCACG | Primers for gene-specific amplification (1^st^ PCR) designed for RF cloning of pCold-II-SUMO |
| pCold-MBP-F | CCATGAATCACAAAGTGCATCATCATCATCATCATAAAACTGAAGAAGGTAAACTGGTAA | Primers for gene-specific amplification (1^st^ PCR) designed for RF cloning of pCold-II-MBP-TEV |
| pCold-MBP-R | CCCCTGGAAGTAAAGGTTCTCGGAAGATCCGGTACCAGTCTGC | Primers for gene-specific amplification (1^st^ PCR) designed for RF cloning of pCold-II-MBP-TEV |
| pCold-Trx-F | CCATGAATCACAAAGTGCATCATCATCATCATCATAGCGATAAAATTATTCACCTGACTG | Primers for gene-specific amplification (1^st^ PCR) designed for RF cloning of pCold-II-TRX-TEV |
| pCold-Trx-R | CCCCTGGAAGTAAAGGTTCTCGGTATGGCCAGAACCAGAACC | Primers for gene-specific amplification (1^st^ PCR) designed for RF cloning of pCold-II-TRX-TEV |
| pCold-II-MZ0064-TFEc_F | CCATGAATCACAAAGTGCATCATCATCATCATCATCAAGTTTCAGTTGAAACCACTCA | Primers for gene-specific amplification (1^st^ PCR) designed for RF cloning of pCold-II-TF-TEV |
| pCold-II-MZ0064-TFEc_R | CGGATCCCTCGAGGGTACCGAGCGCCTGCTGGTTCATCAG | Primers for gene-specific amplification (1^st^ PCR) designed for RF cloning of pCold-II-TF-TEV |
| TF-F | TGGAAGAGCAAGCTGTTGA | Tag-specific primer for sequencing of inserts in pCold-II-TF-TEV constructs |
| MBP-F | GGTCGTCAGACTGTCGATGAAGCC | Tag-specific primer for sequencing of inserts in pCold-II-MBP-TEV constructs |
| MZ0009-RF-F2 | CCGAGAACCTTTACTTCCAGGGGCCGGCAGTGGACCTC | Primers for gene-specific amplification (1^st^ PCR) designed for RF cloning to pCold-II-TEV-based vectors |
| MZ0009-RF-R5 | TCTTAGATTCTGTGCTTTTAAGCAGAGATTACCTAACTGGCTCGCTTATTCTCG | Primers for gene-specific amplification (1^st^ PCR) designed for RF cloning to pCold-II-TEV-based vectors |
| MZ0012-RF-F3 | CCGAGAACCTTTACTTCCAGGGGCCCAGCTTCCGACCGATC | Primers for gene-specific amplification (1^st^ PCR) designed for RF cloning to pCold-II-TEV-based vectors |
| MZ0012-RF-R2 | TCTTAGATTCTGTGCTTTTAAGCAGAGATTACCTAAGTGGCCATAACGGACGC | Primers for gene-specific amplification (1^st^ PCR) designed for RF cloning to pCold-II-TEV-based vectors |
| MZ0013-RF-F3 | CCGAGAACCTTTACTTCCAGGGGTATCACCGCTTTGGCGAG | Primers for gene-specific amplification (1^st^ PCR) designed for RF cloning to pCold-II-TEV-based vectors |
| MZ0013-RF-R2 | TCTTAGATTCTGTGCTTTTAAGCAGAGATTACCTAGACTGCCCCTGAATGCTG | Primers for gene-specific amplification (1^st^ PCR) designed for RF cloning to pCold-II-TEV-based vectors |
| MZ0003-RF-F | CCGAGAACCTTTACTTCCAGGGGCAGCCGCGCGGATTCAAC | Primers for gene-specific amplification (1^st^ PCR) designed for RF cloning to pCold-II-TEV-based vectors |
| MZ0003-RF-R | TCTTAGATTCTGTGCTTTTAAGCAGAGATTACCTATTCTGCACCGCCCGTCAT | Primers for gene-specific amplification (1^st^ PCR) designed for RF cloning to pCold-II-TEV-based vectors |
| MZ0047-RF-F | CCGAGAACCTTTACTTCCAGGGGACCGCCGTTCCCCAACTC | Primers for gene-specific amplification (1^st^ PCR) designed for RF cloning to pCold-II-TEV-based vectors |
| MZ0047-RF-R | TCTTAGATTCTGTGCTTTTAAGCAGAGATTACCTAGAAATCGTCAGGCCTAACGC | Primers for gene-specific amplification (1^st^ PCR) designed for RF cloning to pCold-II-TEV-based vectors |
| pCold-F | ACGCCATATCGCCGAAAGG | Vector-specific primers for sequencing of pCold-II-based plasmids |
| pCold-R | TGGCAGGGATCTTAGATTCTG | Vector-specific primers for sequencing of pCold-II-based plasmids |
| MZ0003-RF-F2 | CTCATCGTGAACAAATCGGCGGTCAGCCGCGCGGATTCAAC | Primers for gene-specific amplification (1^st^ PCR) designed for RF cloning to pCold-II-SUMO |
| MZ0047-RF-F2 | CTCATCGTGAACAAATCGGCGGTACCGCCGTTCCCCAACTC | Primers for gene-specific amplification (1^st^ PCR) designed for RF cloning to pCold-II-SUMO |
| MZ0013-RF-F4 | CTCATCGTGAACAAATCGGCGGTTATCACCGCTTTGGCGAG | Primers for gene-specific amplification (1^st^ PCR) designed for RF cloning to pCold-II-SUMO |
| MZ0012-RF-F5 | CTCATCGTGAACAAATCGGCGGTTTCCGACCGATCATACGC | Primers for gene-specific amplification (1^st^ PCR) designed for RF cloning to pCold-II-SUMO |
| MZ0009-RF-F4 | CTCATCGTGAACAAATCGGCGGTGCAGTGGACCTCGTCGG | Primers for gene-specific amplification (1^st^ PCR) designed for RF cloning to pCold-II-SUMO |

**Supplementary figure labels**

**Figure S1. Alignment of MZ0009 with characterized GH18 chitinases.**

The relevant region containing the substrate binding motif (SXGG) and the catalytic motif (DXDXE, wherein catalytic residues are highlighted with black circles) of MZ0009 and aligned sequences of characterized GH18 chitinases is shown. The label on the left side of each sequence gives its name, UniProt ID [[1](#_ENREF_1)] and organism: SERMA, Serratia marcescens; SACD2, Saccharophagus degradans (strain 2-40); VIBHA, Vibrio harveyi; STRCO, Streptomyces coelicolor. Amino acid numbering is indicated on the left of each sequence. Secondary structure information is retrieved from *S. marcescens* ChiB (UniProt ID: **Q54276**, PDB ID: **1E15**). Dark backgrounds indicate alignment of identical residues, and lined boxes indicate alignment of similar residues. The alignment was constructed using the multiple alignment program ClustalX2.0 [[2](#_ENREF_2)]. Sequence similarities and secondary structure information (stands and helices are shown as black arrows and spirals above the alignment) from aligned sequences was rendered by ESPript3.0 [[3](#_ENREF_3)].

**References**

1. Consortium, T.U., *Activities at the Universal Protein Resource (UniProt).* Nucleic Acids Research, 2014. 42(D1): p. D191-D198.

2. Larkin, M.A., et al., *Clustal W and Clustal X version 2.0.* Bioinformatics, 2007. 23(21): p. 2947-8.

3. Robert, X. and P. Gouet, *Deciphering key features in protein structures with the new ENDscript server.* Nucleic Acids Research, 2014. 42(W1): p. W320-W324.
