# Supplementary figures and images for: Cold shock induction of recombinant Arctic environmental genes
Source: BMC Biotechnol. 2015 Aug 19;15:78. doi: 10.1186/s12896-015-0185-1 (PMC4544801; doi:10.1186/s12896-015-0185-1)

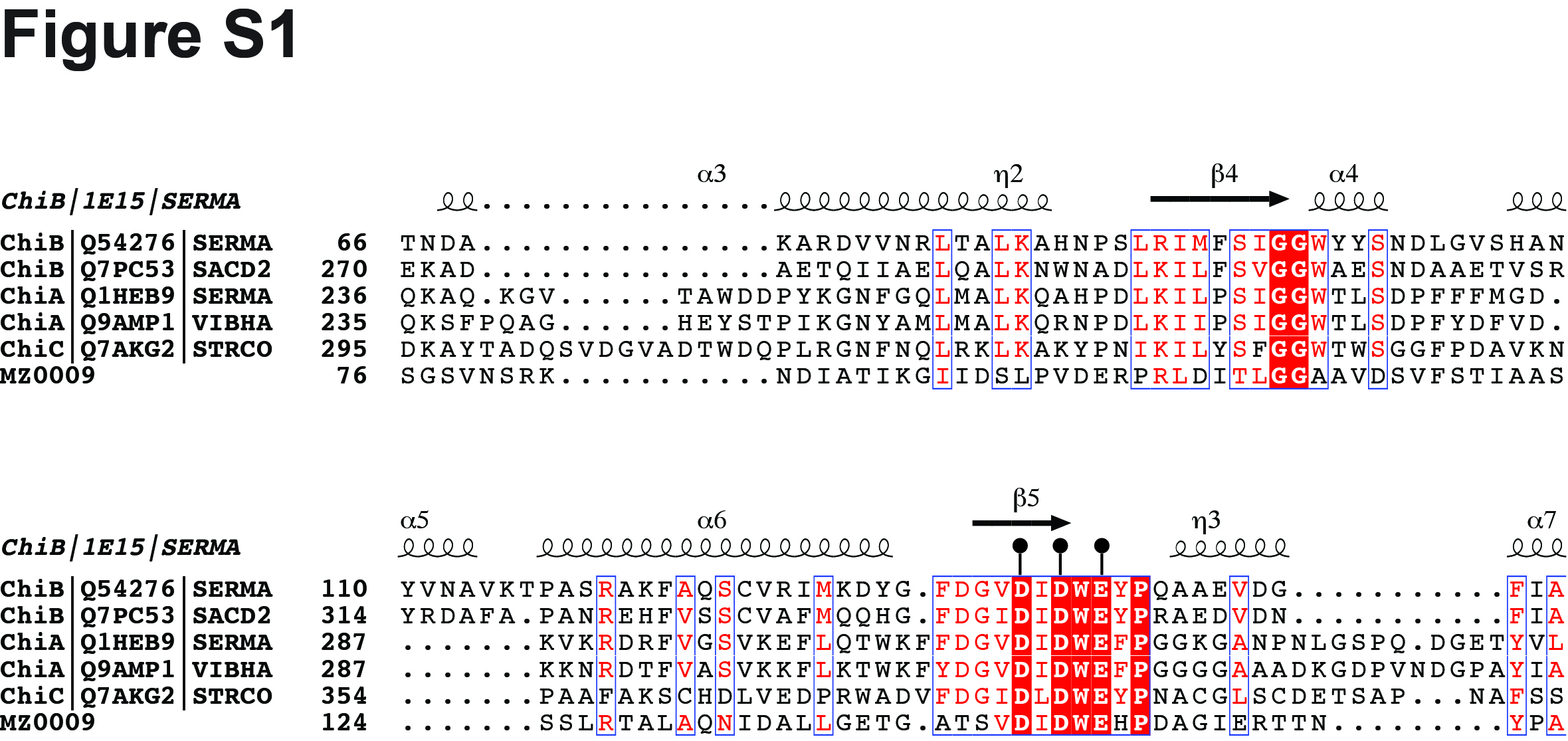

Supplement: Additional file 2: — Sequence alignment of MZ0009 with characterized GH18 chitinases. (JPEG 3038 kb). [file 12896_2015_185_MOESM2_ESM.jpeg]
